# The *Neosartorya fischeri* Antifungal Protein 2 (NFAP2): A New Potential Weapon against Multidrug-Resistant *Candida auris* Biofilms

**DOI:** 10.3390/ijms22020771

**Published:** 2021-01-14

**Authors:** Renátó Kovács, Fruzsina Nagy, Zoltán Tóth, Lajos Forgács, Liliána Tóth, Györgyi Váradi, Gábor K. Tóth, Karina Vadászi, Andrew M. Borman, László Majoros, László Galgóczy

**Affiliations:** 1 Department of Medical Microbiology, Faculty of Medicine, University of Debrecen, Nagyerdei krt. 98, 4032 Debrecen, Hungary; nagyfruzsina0429@gmail.com (F.N.); toth.zoltan@med.unideb.hu (Z.T.); forgacs.lajos.89@gmail.com (L.F.); vadaszikarina13@freemail.hu (K.V.); major@med.unideb.hu (L.M.); 2 Faculty of Pharmacy, University of Debrecen, Nagyerdei krt. 98, 4032 Debrecen, Hungary; 3 Department of Metagenomics, University of Debrecen, Nagyerdei krt. 98, 4032 Debrecen, Hungary; 4 Doctoral School of Pharmaceutical Sciences, University of Debrecen, Nagyerdei krt. 98, 4032 Debrecen, Hungary; 5 Department of Pharmacology and Pharmacotherapy, Faculty of Medicine, University of Debrecen, Nagyerdei krt. 98, 4032 Debrecen, Hungary; 6 Institute of Plant Biology, Biological Research Centre, Temesvári krt. 62, 6726 Szeged, Hungary; toth.liliana@brc.hu (L.T.); galgoczi.laszlo@brc.hu (L.G.); 7 Department of Biotechnology, Faculty of Science and Informatics, University of Szeged, Közép fasor 52, 6726 Szeged, Hungary; 8 Department of Medical Chemistry, Faculty of Medicine, University of Szeged, Dóm tér 8, 6720 Szeged, Hungary; varadi.gyorgyi@med.u-szeged.hu (G.V.); toth.gabor@med.u-szeged.hu (G.K.T.); 9 MTA-SZTE Biomimetic Systems Research Group, University of Szeged, Dóm tér 8, 6720 Szeged, Hungary; 10 UK National Mycology Reference Laboratory, Public Health England, Science Quarter, Southmead Hospital, Bristol BS10 5NB, UK; andy.borman@nbt.nhs.uk; 11 Medical Research Council Centre for Medical Mycology (MRC CMM), University of Exeter, Exeter EX4 4QD, UK

**Keywords:** antifungal lock therapy, *Candida auris*, biofilm, NFAP2, drug–drug interaction, antifungal susceptibility testing

## Abstract

*Candida auris* is a potential multidrug-resistant pathogen able to persist on indwelling devices as a biofilm, which serve as a source of catheter-associated infections. *Neosartorya fischeri* antifungal protein 2 (NFAP2) is a cysteine-rich, cationic protein with potent anti-*Candida* activity. We studied the in vitro activity of NFAP2 alone and in combination with fluconazole, amphotericin B, anidulafungin, caspofungin, and micafungin against *C. auris* biofilms. The nature of interactions was assessed utilizing the fractional inhibitory concentration index (FICI), a Bliss independence model, and LIVE/DEAD viability assay. NFAP2 exerted synergy with all tested antifungals with FICIs ranging between 0.312–0.5, 0.155–0.5, 0.037–0.375, 0.064–0.375, and 0.064–0.375 for fluconazole, amphotericin B, anidulafungin, caspofungin, and micafungin, respectively. These results were confirmed using a Bliss model, where NFAP2 produced 17.54 μM^2^%, 2.16 μM^2^%, 33.31 μM^2^%, 10.72 μM^2^%, and 111.19 μM^2^% cumulative synergy log volume in combination with fluconazole, amphotericin B, anidulafungin, caspofungin, and micafungin, respectively. In addition, biofilms exposed to echinocandins (32 mg/L) showed significant cell death in the presence of NFAP2 (128 mg/L). Our study shows that NFAP2 displays strong potential as a novel antifungal compound in alternative therapies to combat *C. auris* biofilms.

## 1. Introduction

*Candida auris* is the first fungal pathogen to be announced as a global public health threat due to its ability to spread from patient-to-patient and cause invasive infections with high mortality [1,2,3]. Although, the majority of *C. auris* isolates have been recovered from patients with candidemia; several cases have also been observed from catheter-associated infections because *C. auris* shows a potent capacity to develop biofilms on medical devices [4,5,6]. Clinical surveys indicated that catheters were the predominant source of infection in 89% of *C. auris* candidaemia cases while this ratio was only 46% in non-*C. auris* bloodstream infections [7,8]. Recently, it was reported that nearly 40% of clinical *C. auris* isolates exhibit a multidrug-resistant phenotype, which is more pronounced in sessile communities. In addition, the ratio of pan-resistant isolates to all three commonly prescribed antifungal drugs is increasing in multiple countries [9,10].

In the last decade, alternative antifungal strategies, such as antifungal lock therapy-has received more attention as an alternative salvage therapy to eradicate intraluminal *Candida* biofilms [11,12]. To date, there is no officially approved antifungal lock strategy, such an approach would be particularly important in certain populations, such as patients with coagulopathies [13]. Echinocandins are promising targets for several potential lock solutions; however, the minimum inhibitory concentration (MIC_90_) values of *C. auris* biofilms ranged from 0.25 to >32 mg/L for caspofungin and micafungin, which represents a 2- to >512-fold increase in resistance when compared to planktonic cells, a difference that is likely to negatively impact clinical outcome [10,14].

A new potential lock strategy may be the antifungal protein-based lock solution against *C. auris* biofilms; although, to date, there are no data regarding the susceptibility of *C. auris* biofilms to antifungal proteins. *Neosartorya fischeri* antifungal protein 2 (NFAP2) is a novel member of small cysteine-rich and cationic antifungal proteins from filamentous ascomycetes (crAFPs) [15]. Previous studies demonstrated that this protein has a potential applicability in the treatment of *Candida* infections. NFAP2 inhibited the growth of clinically relevant *Candida* species. Furthermore, it interacted synergistically in combination with fluconazole against planktonic and sessile *C. albicans* cells in vitro and in vivo [16,17]. In light of these promising findings, the present study aimed to examine the in vitro efficacy of NFAP2 alone and in combination with traditional antifungal agents against *C. auris* biofilms to evaluate a new potential therapeutic approach against this fungal superbug.

## 2. Results

### 2.1. In Vitro Susceptibility of Planktonic Cells

The median MICs against planktonic *C. auris* cells (pMIC) ranged from 4 to > 32 mg/L, 0.25 to 1 mg/L, 0.06 to 0.5 mg/L, 0.5 to 1 mg/L, and 0.12 to 2 mg/L for fluconazole, amphotericin B, anidulafungin, caspofungin, and micafungin, respectively. The median pMIC to fluconazole of strains 12 and 27 was 4 mg/L, which correspond to the susceptible tentative breakpoint; while isolates 10, 20, and 82 were considered fluconazole-resistant based on the tentative MIC breakpoints recommended by the Centers for Disease Control and Prevention (≥32 mg/L for fluconazole) [18]. Regarding amphotericin B and the tested echinocandins, all isolates were susceptible according to tentative MIC breakpoints (≥2 mg/L for amphotericin B, ≥4 mg/L for anidulafungin, ≥2 mg/L for caspofungin, and ≥4 mg/L for micafungin) [18]. The median pMICs for NFAP2 ranged from 32 to 512 mg/L.

### 2.2. In Vitro Susceptibility of Sessile Biofilm Cells

The MIC results (medians and ranges) on *C. auris* biofilms (sMIC) are shown in Table 1. Most of the isolates in biofilm form proved to be resistant to fluconazole, anidulafungin, caspofungin, micafungin, and NFAP2; while amphotericin B effectively inhibited the viability of sessile *C. auris* cells (sMIC: 1–2 mg/L) (Table 1). The median sMICs observed for fluconazole, amphotericin B, anidulafungin, caspofungin, and micafungin in combination with NFAP2 were reduced by 32- to 128-fold, 4- to 64-fold, 16- to 128-fold, 4- to 128-fold, and 64- to 128-fold, respectively (Table 1). The median sMICs for NFAP2 exhibited a 2- to 4-fold, 4- to 128-fold, 8- to 128-fold, 8- to 16-fold, and 4- to 256-fold decrease combined with fluconazole, amphotericin B, anidulafungin, caspofungin, and micafungin, respectively (Table 1). These results indicated that the combinatorial application of the tested conventional antifungal agents with NFAP2 results in a significant reduction in the viability of sessile cells.

### 2.3. Nature of the NFAP2-Antifungal Drugs Interactions

**Figure 1:**
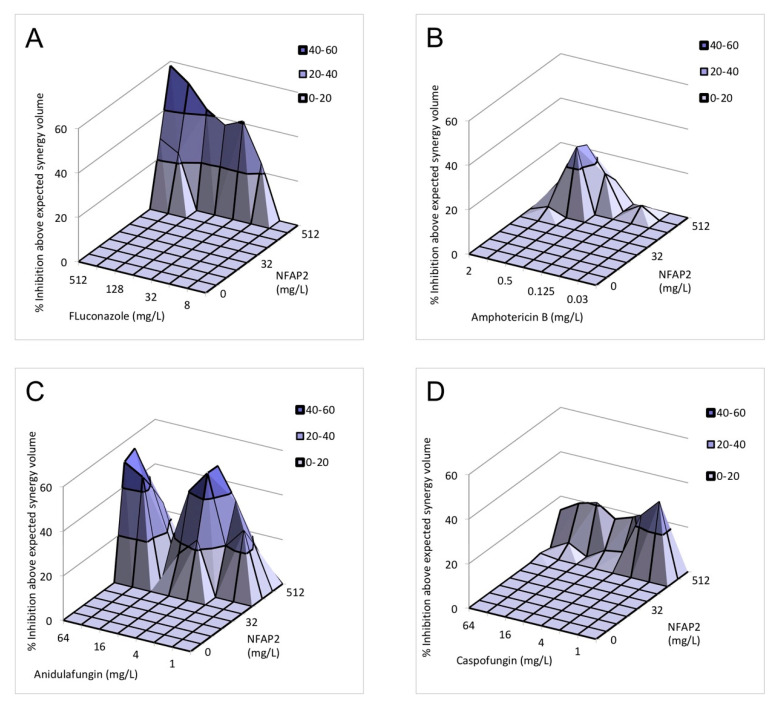

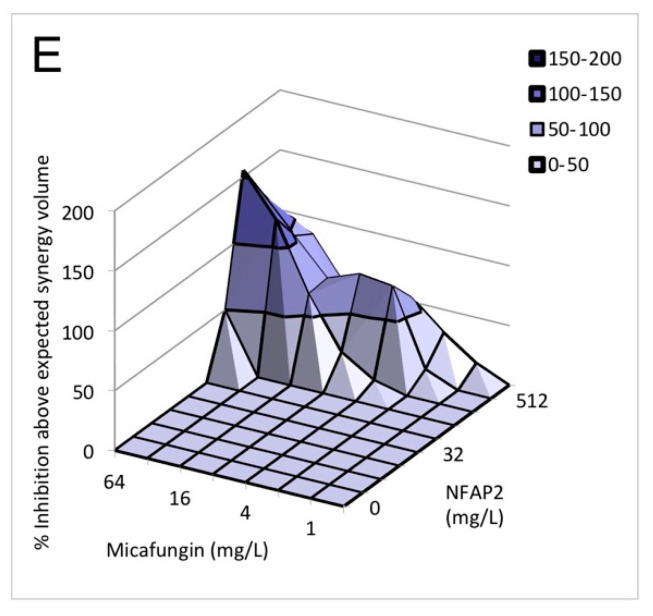
Effect of NFAP2 in combination with fluconazole (**A**), amphotericin B (**B**), anidulafungin (**C**), caspofungin (**D**) and micafungin (**E**) against *C. auris* isolates using MacSynergy II analysis. Additive interactions appear as a horizontal plane at 0% inhibition. The interaction is defined as synergistic if the observed surface is greater compared to the predicted additive surface. The volumes are calculated at the 95% confidence interval. The figures represent the cumulative synergy volume of five tested isolates. In panel E, higher synergy was observed; therefore, the scale of the *z* axis is different than in panels (**A**–**D**). Each figure presents the cumulative values of the five tested *C. auris* isolates.

Table 2 summarises the nature of in vitro interactions between NFAP2 and the five tested antifungal drugs based on the calculated fractional inhibitory concentration index (FICI). Synergy was observed for all antifungals and all isolates. Median FICI values ranged from 0.312 to 0.5, 0.155 to 0.5, 0.037 to 0.375, 0.064 to 0.375, and 0.064 to 0.375 for fluconazole, amphotericin B, anidulafungin, caspofungin, and micafungin, respectively (Table 2). The results obtained by FICI were partly confirmed using a Bliss independence model. It is noteworthy, that strain dependency of the nature of the drug interaction was observed when the strains were tested individually using MacSynergy II analysis (Table 2), which was prominent at lower concentrations of the combined antifungal drugs. However, this strain dependency disappeared when the five strains were analysed simultaneously. This cumulative analysis indicated that NFAP2 exerts 17.54 μM^2^%, 2.16 μM^2^%, 33.31 μM^2^%, 10.72 μM^2^%, and 111.19 μM^2^% cumulative synergy volume in combination with fluconazole, amphotericin B, anidulafungin, caspofungin and micafungin, respectively (Table 2 and Figure 1).

### 2.4. Fluorescence Viability Assay

**Figure 2:**
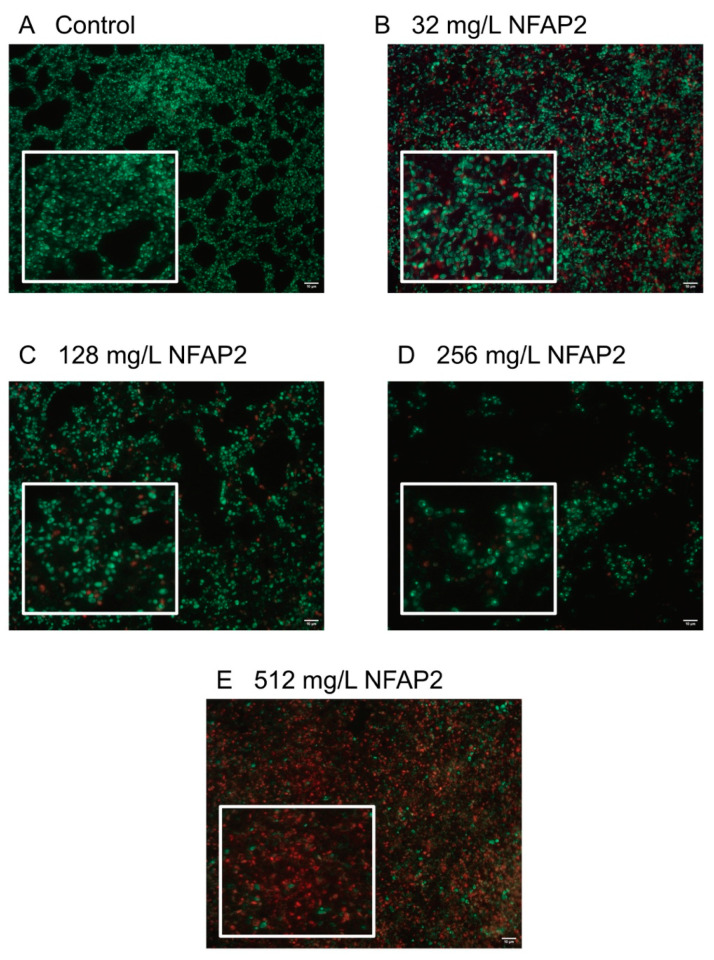
LIVE/DEAD fluorescence imaging of one representative *C. auris* isolate (isolate 10). Image (**A**) shows the untreated biofilm, while images (**B**–**E**) present the NFAP-exposed biofilms at 32 mg/L, 128 mg/L, 256 mg/L and 512 mg/L NFAP2 concentrations, respectively. Live cells (green) and nonviable cells (red) were stained with Syto9 and propidium iodide, respectively. All images show typical fields of view. Scale bars represent 10 μm.

**Figure 3:**
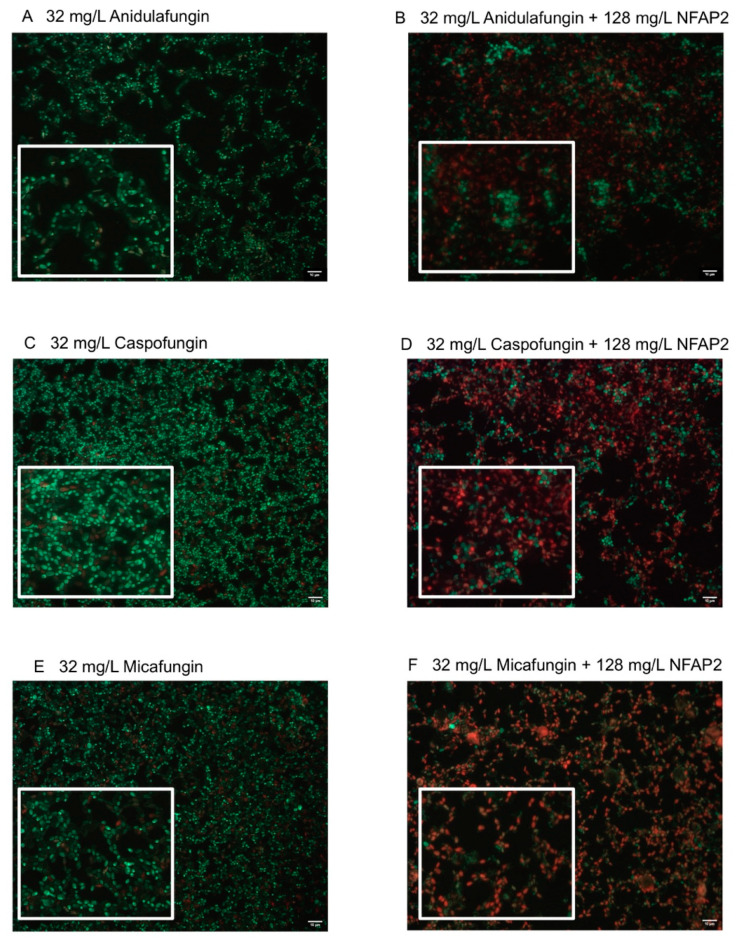
LIVE/DEAD fluorescence imaging of one representative *C. auris* isolate (isolate 10). Images (**A**,**C**,**E**) demonstrate the anidulafungin-, caspofungin- and micafungin-exposed biofilms (32 mg/L), respectively, while images (**B**,**D**,**F**) show the anti-biofilm effect of anidulafungin, caspofungin and micafungin (32 mg/L for each drug alone) in the presence of NFAP2 (128 mg/L), respectively. Live cells (green) and nonviable cells (red) were stained with Syto9 and propidium iodide, respectively. All images show typical fields of view. Scale bars represent 10 μm.

The LIVE/DEAD viability assay focused primarily on the echinocandins because this antifungal group is considered in alternative anti-biofilm therapeutic strategies, such as antifungal lock therapy [11]. For the NFAP2 concentrations tested (Figure 2A–E), an extensive anti-biofilm effect was only observed after exposure to 512 mg/L NFAP2 (Figure 2E), when compared to untreated one-day old biofilms (Figure 2A). The NFAP2 treatment alone showed a concentration-dependent activity, where the ratio of dead cells was 9%, 43%, 67% and 90% after 32 mg/L, 128 mg/L, 256 mg/L and 512 mg/L NFAP2 exposure, respectively (Figure 2A–E). It is noteworthy that the 128 mg/L NFAP2 (Figure 2C) and 32 mg/L echinocandin treatments alone did not produce remarkable cell death. The ratio of dead cells was 28%, 16% and 24% in the samples following the 32 mg/L anidulafungin, caspofungin and micafungin treatments, respectively (Figure 3A,C,E). However, their combined application with 128 mg/L NFAP2 resulted in a significant total cell number reduction. The cell number decreased with 44%, 34% and 41% after co-application of 128 mg/L NFAP2 with 32 mg/L anidulafungin, caspofungin and micafungin, respectively (Figure 3B,D,F). In addition, the percentage of dead cells was 74%, 72% and 60% in these samples, respectively (Figure 3B,D,F). This observation further strengthened the results of the previous XTT assay, and clearly demonstrates that co-administration of NFAP2 with echinocandins on potentially echinocandin-resistant biofilm rendered them susceptible to these antifungal agents.

## 3. Discussion

The eradication of *C. auris* biofilms from medical indwelling devices (e.g., catheters and cannulas) still remains a big challenge in the nosocomial environment because these sessile communities can withstand exposure to the most frequently administered antifungal agents. Thus, biofilms serve as a continuous source of *C. auris*-related candidaemia [8]. Currently, several novel antifungal drugs are under development against *C. auris*, including ibrexafungerp [19], manogepix [20], VT-1598 [21], and rezafungin [22] and they may represent potential treatment options in the near future. However, considering the increasing number of multidrug-resistant *C. auris* isolates, new and alternative therapeutic strategies are needed to prevent and eliminate the growth of *C. auris* biofilms from indwelling devices.

Although there is increasing recognition that antibiotic lock solutions can reduce the risk of catheter-related bacterial infections, there is no approved antifungal lock therapeutic protocol in clinical practice so far. Vargas-Cruz et al. (2019) reported that liposomal amphotericin B, amphotericin B deoxycholate, fluconazole, voriconazole, micafungin, caspofungin, and anidulafungin failed to completely eradicate *C. auris* biofilms, contraindicating their mono-therapeutic use in lock therapy [23]. Therefore, certain non-antifungal agents alone or in combination with traditional antifungals have been investigated as a potential approach to overcome *C. auris*-related catheter-associated infections. These include ebselen [24], miltefosine [25], farnesol [26], and silver or bismuth nanoantibiotics [27,28]. To date, three conventional potential line lock solutions have been tested against *C. auris* biofilms. Taurolidine showed moderate activity against biofilms and only partially eradicated sessile populations. Conversely, the minocycline-EDTA-ethanol lock solution and nitroglycerin-citrate-ethanol combination completely eradicated *C. auris* biofilms in vitro [23,29].

The environmentally highly stable crAFPs represent promising bioactive natural compounds in anti-*Candida* therapy [30], and they can provide potential bases to develop new lock solutions against *Candida* biofilms. Although the number of these molecules is steadily increasing, only a few of them have been well-characterised so far [16,17,30]. It is noteworthy that several cell-culture-based cytotoxicity assays proved that crAFPs have no remarkable cytotoxic effects on mammalian cells in vitro and in vivo. Additionally, physiologically compatible solutions can be prepared from the majority of these proteins, which is a basic requirement for a lock solution [30]. Although novel therapeutic approaches focusing on the activity of short antifungal peptides from other origins against *C. auris* have previously been published, the potential effects of crAFPs remained unknown.

Del Mas et al. (2019) demonstrated the effect of crotamine, the venom of South American rattlesnake, which exerted 50% inhibition of *C. auris* planktonic growth at a concentration of 160 μM [31]. Van Eijk et al. (2020) described that two cathelicidin-inspired antimicrobial peptides (CT172 and CR 184) strongly interfered with metabolic activity, growth, and viability at sub-micromolar levels (≤1 μM) against *C. auris* planktonic cells [32]. Kubiczek et al. (2020) reported that antifungal peptides might have a promising anti-biofilm activity: derivates of the antifungal peptide Cm-p5 exhibited a semi-inhibitory effect at concentrations ranging from 10 to 21 mg/L against *C. auris* biofilms. In addition, the mature sessile populations were also inhibited by 71–97% [33].

NFAP2 represents a novel, phylogenetically distinct group of crAFPs [15]. *In silico* analysis predicted that NFAP2 has a strong ability to bind human serum albumin, questioning the systemic application of this compound [17]. Nevertheless, it may be a promising target in the above-mentioned newly defined antifungal lock strategies. The previously well-documented membrane disrupting effect renders NFAP2 suitable as very potent anti-*Candida* compound [17]. Before the present study, an in vitro synergistic interaction was already documented between NFAP2 and fluconazole against *C. albicans* and *C. parapsilosis*, suggesting the justification of NFAP2 in combination-based therapies [16]. Furthermore, the in vivo therapeutic potency of NFAP2 as a topical agent was proven for the treatment of vulvovaginal candidiasis caused by fluconazole-resistant *C. albicans* in a murine model system [17]. Kovács et al. (2019) reported that 800 mg/L daily NFAP2 together with 5 mg/kg daily fluconazole treatment was superior compared to 5 mg/kg daily fluconazole treatment. In addition, this NFAP2 concentration did not cause significant morphological alterations in the vaginal and vulvar tissues and did not show a cytotoxic effect on human keratinocytes and dermal fibroblasts in vitro [17]. Synergistic interaction between NFAP2 and fluconazole were already reported against *C. albicans* [16]. In the present study, NFAP2 alone showed a concentration-dependent activity against *C. auris* biofilms (Figure 2). Based on FICI calculation, synergism was detected for NFAP2 in the presence of fluconazole, amphotericin B, anidulafungin, caspofungin, and micafungin against *C. auris* biofilms (Table 2). It is noteworthy that the synergistic interaction was observed primarily at high NFAP2 concentrations ranging between 4–512 mg/L (Table 1). The interaction between NFAP2 and the tested antifungals was variable based on the Bliss independence model (Table 2). This strain dependency was observed primarily at lower concentrations of the combined drugs and was not observed at their higher concentrations. Furthermore, this variability disappeared when the five strains were analysed simultaneously by MacSynergy II algorithm. Existence of such variability points to the necessity to use multiple analytic approaches in parallel when examining drug–drug interactions.

The mechanism underlying the synergy observed between NFAP2 and the antifungals tested here might result from the fact that NFAP2 has a pore-forming effect in the cell membrane [17], which would exacerbate the osmotic stress derived from echinocandin-related cell wall damage and from membrane-active antifungals, such as fluconazole and amphotericin B. Our study had a limitation: we examined strains derived from only one *C. auris* clade (South Asian/Indian lineage). However, despite this limitation, the potentiator effect of NFAP2 in combination with traditional antifungals against *C. auris* one-day-old biofilms is unquestionable.

In summary, improvements and clinical verifications in alternative combination-based antifungal therapies can help the development of new treatment strategies against *C. auris* biofilms. Based on our in vitro findings, combined application of NFAP2 with widely used traditional antifungals may provide a potential novel approach in the antifungal armoury of *C. auris*-specific alternative treatments as lock therapy. In the future, further animal experiments of these new combinations are warranted.

## 4. Materials and Methods

### 4.1. Isolates

Five *C. auris* isolates (isolate 10, 12, 20, 27, and 82) derived from the South Asian/Indian lineage were obtained from the National Mycology Reference Laboratory, United Kingdom. Strain 10 (NCPF 8971) and 20 (NCPF 8985) were isolated from wound swabs. Isolate 27 (NCPF 89891) and 82 (NCPF 13013) were obtained from pleural fluid and urine, respectively, while the source of strain 12 (NCPF 8973) was not stated [34,35]. Each strain derived from different patients. All isolates were identified to species level by Matrix-Assisted Laser Desorption-Ionisation-Time of Flight Mass Spectrometry [34,35]. Clade delineation was conducted by PCR amplification and sequencing of the 28S rDNA gene and the internal transcribed spacer region 1, as described previously [34,35].

### 4.2. Recombinant NFAP2 Production and Purification

Recombinant NFAP2 was produced in a *Penicillium chrysogenum*-based expression system and purified to 100% homogeneity, as described previously by Kovács et al. (2019) [17]).

### 4.3. In Vitro Susceptibility Testing of Planktonic Cells

pMIC were determined in line with the protocol M27-A3 of the Clinical Laboratory Standards Institute [36]. pMICs of fluconazole (cat. # J62015, VWR, Debrecen, Hungary), amphotericin B (cat. # Y0001361, Merck, Budapest, Hungary) anidulafungin (cat. # ADF00-100, Molcan Corporation, Toronto, ON, Canada), caspofungin (cat. # CSF00A-100, Molcan Corporation, Toronto, ON, Canada), micafungin (cat. # MCF00N-100, Molcan Corporation, Toronto, ON, Canada) and NFAP2 were determined in RPMI 1640 (with L-glutamine and without bicarbonate, pH 7.0 with MOPS; Merck, Budapest, Hungary). The drug concentrations tested ranged from 0.06 to 32 mg/L, 0.008 to 4 mg/L, and 0.008 to 4 mg/L for fluconazole, amphotericin B, and echinocandins, respectively; while NFAP2 concentrations ranged from 1 to 512 mg/L (corresponding to 0.2–92 μM). For fluconazole, echinocandins and NFAP2, pMICs were determined as the lowest drug concentration that produces at least 50% growth reduction compared to the growth of the control. For amphotericin B, pMIC was considered the first concentration exerting 100% growth inhibition compared to the growth of the drug-free control. pMICs represent three independent experiments for each isolate and are presented as the median.

### 4.4. Biofilm Development

One-day-old biofilms were prepared as described in our previous studies [26,37,38]. Briefly, *C. auris* isolates were suspended in RPMI 1640 liquid medium to a final concentration of 1 × 10^6^ cells/mL, and aliquots of 0.1 mL were pipetted onto flat-bottom 96-well sterile microtiter plates (TPP, Trasadingen, Switzerland) and then incubated statically at 37 °C for 24 h. After the incubation time, plates were washed three times with physiological saline to remove unattached cells.

### 4.5. Antifungal Susceptibility Testing of Biofilms

The concentrations tested for sMIC determination ranged from 8 to 512 mg/L, 0.03 to 2 mg/L, and 1 to 64 mg/L for fluconazole, amphotericin B, and echinocandins, respectively. Meanwhile, the examined NFAP2 concentrations ranged from 2 to 512 mg/L. The prepared one-day-old biofilms were washed three times with sterile physiological saline. Different drug concentrations in RPMI 1640 were added to one-day-old pre-formed biofilms and then the plates were incubated for an additional 24 h at 37 °C. Afterwards, sMIC determinations were carried out using the metabolic activity change-based XTT [2,3-bis(2-methoxy-4-nitro-5-sulfophenyl)-2*H*-tetrazolium-5-carboxanilide] reduction assay. The prepared XTT working solution (Merck, Budapest, Hungary) (0.5 g/L) was supplemented with menadione (Merck, Budapest, Hungary) to a final concentration of 1 μM [26,37,38,39]. Drugs were removed prior to assay of metabolic activity by washing three times with sterile physiological saline. Afterwards, a 100 μL aliquot of XTT/menadione solution was added to each well containing the preformed biofilms as well as to negative control wells. Plates were incubated in darkness for 2 h at 37 °C. Following incubation, 80 μL of supernatant from each well was measured spectrophotometrically at 492/620 nm (Multiskan Sky Microplate Spectrophotometer, Thermo-Scientific^TM^, Waltham, MA, USA). sMICs were considered as the lowest drug concentration exerting at least a 50% reduction in metabolic activity compared to the untreated biofilms [26,37,38,39]. sMICs represent three independent experiments for each isolate and are expressed as the median value.

### 4.6. Evaluation of Interactions by Fractional Inhibitory Concentration Index (FICI) and Bliss Independence Model

Interactions between tested antifungal agents and NFAP2 were evaluated using a previously well-documented two-dimensional broth microdilution checkerboard assay [26,37,40]. Antifungal drug-NFAP2 interactions were then analyzed using FICI determination and a Bliss independence model-based MacSynergy II analysis [26,37,38,39,40,41,42]. The tested concentration ranges were the same as those described in the previous section for sMIC determination. FICIs were calculated using the following formula: ΣFIC = FIC_A_ + FIC_B_ = MIC_A_^comb^/MIC_A_^alone^ + MIC_B_^comb^/MIC_B_^alone^, where MIC_A_^alone^ and MIC_B_^alone^ represent the MICs of drugs A and B when used alone, and MIC_A_^comb^ and MIC_B_^comb^ are the MIC of drugs A and B in combination at isoeffective combination, respectively [40]. FICI was determined as the lowest ΣFIC. sMIC values of the tested antifungals and NFAP2 alone and of all isoeffective combinations were determined as the lowest concentration, resulting in at least a 50% decrease in metabolic activity compared to the growth control sessile cells. If the obtained MIC value was higher than the highest tested drug concentration, the next highest two-fold concentration was considered as the MIC. The obtained FICIs were interpreted based on the following algorithm: synergistic interaction was defined as FICI ≤ 0.5, an indifferent interaction as a FICI between >0.5 and 4, and antagonistic interaction as FICI >4 [26,37,40]. FICIs were determined in three independent experiments, and their median values were presented with ranges.

To further evaluate antifungal drugs–NFAP2 interactions, MacSynergy II analysis was used in case of all isolates, employing the Bliss independence algorithm in a Microsoft Excel-based interface to determine synergy. MacSynergy-based analysis was performed as previously described [26,37,41,42]. Briefly, synergy and antagonistic volumes were calculated by adding all of the positive values and all of the negative values for each drug combination, respectively [26,37,41,42]. These volumes were then statistically evaluated using the 95% confidence level and expressed in units of μM^2^%, which are analogous to the units for area under a dose–response curve in the two-dimensional graph [26,37,41,42]. Synergy or antagonism is significant if the interaction volumes are >25 μM^2^% or <25 μM^2^%, respectively (corresponding to log volumes > 2 and log volumes < 2, respectively). Values between 25 μM^2^% and 50 μM^2^% (values in log volume between >2 to 5) should be considered as minor synergy. Values between 50 μM^2^% and 100 μM^2^% (values in log volume between >5 to 9) indicate moderate synergy or antagonism, while values over 100 μM^2^% (values in log volume between >9) represent strong synergy [26,37,41,42]. When a small number of drug concentration combinations results in antagonistic interaction in a generally synergistic combination, the applied terminology is ‘synergy for most combinations’. While a small number of drug concentration combinations results in synergistic interaction in a generally antagonistic combination, the applied terminology is ‘antagonism for most combinations’ [41].

### 4.7. Biofilm Viability Assay in the Presence or Absence of NFAP2

*C. auris* biofilms were grown on the surface of 8-well Permanex slides statically at 37 °C for 24 h (Lab-Tek^®^ Chamber Slide™ System, VWR, Debrecen, Hungary) [26,43]. The one-day-old biofilms were washed three times with physiological saline. After the washing step, the antifungal effect of NFAP2 (32 mg/L, 128 mg/L, 256 mg/L, and 512 mg/L) and echinocandins (32 mg/L) alone, and echinocandins (32 mg/L)-NFAP2 (128 mg/L) combinations were tested on the sessile biofilm cells. These concentrations were chosen based on our previous antifungal susceptibility test results on biofilms.

Following 24 h of drug exposure statically at 37 °C, biofilms were washed with sterile physiological saline, then the ratio of viable and dead cells was evaluated using the fluorescent LIVE/DEAD^®^ BacLight™ viability kit (ThermoFisher Scientific, Waltham, MA, USA). Biofilms were stained for 15 min in darkness at 37 °C using Syto 9 (3.34 mM solution in DMSO) and propidium iodide (20 mM solution in DMSO) to visualize viable and non-viable *C. auris* cells, respectively [26,43]. Fluorescent cells were examined with a Zeiss AxioSkop 2 mot microscope (Jena, Germany) coupled with a Zeiss AxioCam HRc camera (Jena, Germany). Axiovision 4.8.2 software was used to analyze images (Jena, Germany). Further picture analysis and calculation of the percentage of the dead cells was performed using ImageJ software (version: 2.1.0/1.53c) (Fiji, ImageJ, Wayne Rasband National Institutes of Health). All images were changed to 8-bit grayscale with background noise subtracted, afterwhich the threshold was defined [44,45].

## Figures and Tables

**Table 1:** Sessile minimum inhibitory concentrations (sMICs) of fluconazole (FLU), amphotericin B (AMB), anidulafungin (ANI), caspofungin (CAS) and micafungin (MICA) alone and in combination with NFAP2 against *C. auris* biofilms.

Drug	Isolate	Median MIC (Range) of Drug Used (50% OD_492_ Reduction in Metabolic Activity)			
		Alone		In Combination	
		Drug (mg/L)	NFAP2 (mg/L)	Drug (mg/L)	NFAP2 (mg/L)
FLU	10	>512 ^a^	>512 ^a^	32	256 (256–512)
	12	>512 ^a^	>512 ^a^	16 (16–32)	256
	20	>512 ^a^	>512 ^a^	32 (32–64)	256 (256–512)
	27	>512 ^a^	>512 ^a^	8 (8–16)	512 (256–512)
	82	>512 ^a^	>512 ^a^	32 (32–128)	512
AMB	10	1	512 (256–512)	0.25	128
	12	1	>512 ^a^	0.03 (0.03–0.06)	64 (64–128)
	20	1	>512 ^a^	0.03 (0.03–0.06)	16 (16–64)
	27	1	512	0.25	64
	82	2	512	0.03 (0.03–0.06)	4 (4–16)
ANI	10	16 (16–32)	>512 ^a^	1	128 (128–256)
	12	>64 ^b^	>512 ^a^	1	32
	20	>64 ^b^	>512 ^a^	1	128 (128–256)
	27	16	512	1	4 (4–8)
	82	>64 ^b^	512	1	64 (64–128)
CAS	10	>64 ^b^	512 (128–512)	32	32 (32–64)
	12	>64 ^b^	>512 ^a^ (512–>512)	1 (1–2)	64
	20	>64 ^b^	512 (256–512)	1	32 (32–64)
	27	>64 ^b^	512 (128–512)	32	32 (32–64)
	82	>64 ^b^	512 (256–512)	1 (1–4)	32
MICA	10	>64 ^b^	>512 ^a^	2 (2–4)	256
	12	>64 ^b^	>512 ^a^	1	4
	20	>64 ^b^	>512 ^a^	1	256 (128–256)
	27	>64 ^b^	>512 ^a^	1	4
	82	>64 ^b^	>512 ^a^	1	32 (32–64)

^a^ MIC is offscale at >512 mg/L, 1024 mg/L (one dilution higher than the highest tested concentration) was used for analysis; ^b^ MIC is offscale at >64 mg/L, 128 mg/L (one dilution higher than the highest tested concentration) was used for analysis.

**Table 2:** In vitro interactions by Fractional Inhibitory Concentration Indexes (FICI) and MacSynergy II analysis of fluconazole (FLU), amphotericin B (AMB), anidulafungin (ANI), caspofungin (CAS) and micafungin (MICA) in combination with *Neosartorya fischeri* antifungal protein 2 (NFAP2) against *C. auris* biofilms.

Drug	Isolate	FICI		MacSynergy II Analysis			
		Median (Range) of FICI	Interaction	Individual Log Volume of Isolates, (Synergy/Antagonism μM^2^%)	Interaction Based on Individual Log Volume	Cumulative Log Volume of Five Isolates, (Synergy/Antagonism μM^2^%)	Interaction Based on Cumulative Log Volume
**FLU**	10	0.5 (0.5–0.53)	Synergy	72.43/−126.35	Antagonism for most combinations	17.54/0	Synergy
	12	0.312	Synergy	12.31/0	Synergy		
	20	0.312 (0.312–0.375)	Synergy	210.17/−3.67	Synergy for most combinations		
	27	0.375 (0.375–0.625)	Synergy	70/−78.26	Antagonism for most combinations		
	82	0.375 (0.375–0.625)	Synergy	119.31/−11.47	Synergy for most combinations		
**AMB**	10	0.5 (0.5–0.75)	Synergy	53.9/−42.3	Synergy for most combinations	2.16/0	Synergy
	12	0.312 (0.312–0.375)	Synergy	196.99/−31.39	Synergy for most combinations		
	20	0.155 (0.155–0.185)	Synergy	126.38/−4.52	Synergy for most combinations		
	27	0.375	Synergy	46.51/−27.26	Synergy for most combinations		
	82	0.25	Synergy	75.39/−12.25	Synergy for most combinations		
**ANI**	10	0.375 (0.25–0.5)	Synergy	127.59/−18.52	Synergy for most combinations	33.31/0	Synergy
	12	0.037	Synergy	371.84/−2.43	Synergy for most combinations		
	20	0.185 (0.185–0.312)	Synergy	154.33/−40.86	Synergy for most combinations		
	27	0.069 (0.069–0.09)	Synergy	71.52/−10.83	Synergy for most combinations		
	82	0.312 (0.185–0.312)	Synergy	148.15/−7.3	Synergy for most combinations		
**CAS**	10	0.375 (0.375–0.75)	Synergy	8.64/−33.23	Antagonism for most combinations	10.72/0	Synergy
	12	0.067 (0.067–0.14)	Synergy	235.24/0	Synergy		
	20	0.075 (0.075–0.257)	Synergy	22.23/−17.48	Synergy for most combinations		
	27	0.375 (0.375–0.75)	Synergy	14.76/−44.63	Antagonism for most combinations		
	82	0.122	Synergy	20.2/−11.59	Synergy for most combinations		
**MICA**	10	0.375	Synergy	164.37/−99.58	Synergy for most combinations	111.19/0	Synergy
	12	0.064	Synergy	277.54/0	Synergy		
	20	0.375	Synergy	378.15/−17.51	Synergy for most combinations		
	27	0.253	Synergy	100.94/−41.11	Synergy for most combinations		
	82	0.132	Synergy	212.19/−18.37	Synergy for most combinations		

## Data Availability

Not applicable.
